# Congenital Adrenal Hyperplasia Causing Poor Response to Androgen Deprivation Therapy in Prostate Cancer

**DOI:** 10.1210/jendso/bvaa158

**Published:** 2020-10-23

**Authors:** Mustafa Kinaan, Oksana Hamidi, Hanford Yau, Kevin D Courtney, Akin Eraslan, Kenneth Simon

**Affiliations:** 1 Department of Internal Medicine, University of Central Florida College of Medicine, Orlando, Florida; 2 Orlando VA Medical Center, Division of Endocrinology, Orlando, Florida; 3 Division of Endocrinology and Metabolism, UT Southwestern Medical Center, Dallas, Texas; 4 Simmons Comprehensive Cancer Center, Department of Internal Medicine, Division of Hematology-Oncology, University of Texas Southwestern Medical Center, Dallas, Texas; 5 Orlando VA Medical Center, Division of Oncology, Orlando, Florida

**Keywords:** congenital adrenal hyperplasia, prostate cancer, hyperandrogenemia, androgen deprivation therapy, adrenal androgens

## Abstract

Androgen deprivation therapy (ADT) is recommended for the treatment of advanced prostate cancer. Inadequate suppression of testosterone while on ADT poses a clinical challenge and requires evaluation of multiple potential causes, including adrenal virilizing disorders. We present 2 cases of elderly patients with prostate cancer who had undiagnosed congenital adrenal hyperplasia (CAH) driving persistent testosterone elevation during ADT. The first patient is a 73-year-old man who underwent radical prostatectomy on initial diagnosis and was later started on ADT with leuprolide following tumor recurrence. He had a testosterone level of 294.4 ng/dL and prostate-specific antigen (PSA) level of 17.7 ng/mL despite leuprolide use. Additional workup revealed adrenal nodular hyperplasia, elevated 17-hydroxyprogesterone (19 910 ng/dL) and dehydroepiandrosterone sulfate (378 mcg/dL), and 2 mutations of the *CYP21A2* gene consistent with simple virilizing CAH. The second patient is an 82-year-old man who received stereotactic radiation therapy at time of diagnosis. He had insufficient suppression of testosterone with evidence of metastatic disease despite treatment with leuprolide and subsequently degarelix. Laboratory workup revealed elevated 17-hydroxyprogesterone (4910 ng/dL) and dehydroepiandrosterone sulfate (312 mcg/dL). Based on clinical, radiographic and biochemical findings, the patient was diagnosed with nonclassic CAH. The first patient initiated glucocorticoid therapy, and the second patient was treated with the CYP17 inhibitor abiraterone in combination with glucocorticoids. Both patients experienced rapid decline in testosterone and PSA levels. Inadequate testosterone suppression during ADT should trigger evaluation for causes of persistent hyperandrogenemia. CAH can lead to hyperandrogenemia and pose challenges when treating patients with prostate cancer.

Androgen deprivation therapy (ADT) is a component of the standard treatment for men with regionally localized high-risk or metastatic prostate cancer [1]. ADT is associated with delayed disease progression and survival benefit [2-4].

Congenital adrenal hyperplasia (CAH) due to 21-hydroxylase enzyme deficiency is an autosomal recessive disorder affecting the adrenal cortex. Impaired conversion of 17-hydroxyprogesterone to 11-deoxycortisol leads to impaired cortisol synthesis. The lack of negative feedback in the hypothalamic-pituitary-adrenal axis due to cortisol deficiency leads to increased adrenocorticotropic hormone (ACTH) levels, which in turn results in stimulation and hyperplasia of the adrenal cortex. The diversion of cortisol precursors to adrenal androgens can cause virilization in affected girls [5, 6]. The severity of the disease correlates closely with the degree of enzyme dysfunction. Simple virilizing form of CAH usually has approximately 1% to 2% of preserved 21-hydroxylase enzyme function and without newborn screening programs can be unrecognized until rapid growth and accelerated skeletal maturation is observed in later childhood, leading to compromised adult stature. Nonclassic (late-onset) CAH (NCCAH) is a less severe form of the disorder with about 5% to 20% of 21-hydroxylase enzyme activity. The degree of hyperandrogenemia is more moderate than that seen in patients with classic CAH. Although usually asymptomatic, NCCAH can present later in life with signs of androgen excess. Androgen excess in males can lead to premature puberty, acne, advanced bone age, short stature, and infertility.

Although the prevalence of classic CAH is rare, with a worldwide incidence of 1 in 14 000 to 18 000 births, NCCAH is one of the most common autosomal recessive diseases reported in 1 in 1000 individuals in the general White population [7], with even higher prevalence among certain ethnic groups [8, 9].

We describe 2 cases of patients diagnosed with CAH following inadequate suppression of testosterone with ADT for prostate cancer.

## Case Presentation

### Patient 1

**Figure 1.**
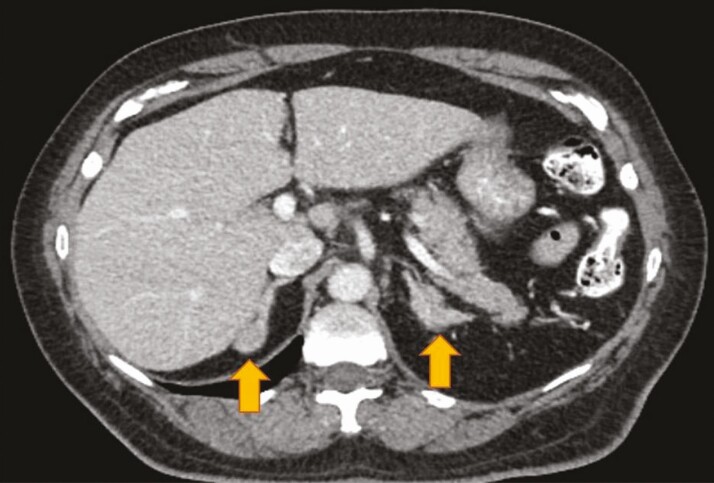
Cross-sectional abdominal computed tomography shows bilateral adrenal nodular hyperplasia (arrows) in Patient 1 with simple virilizing congenital adrenal hyperplasia.

**Figure 2.**
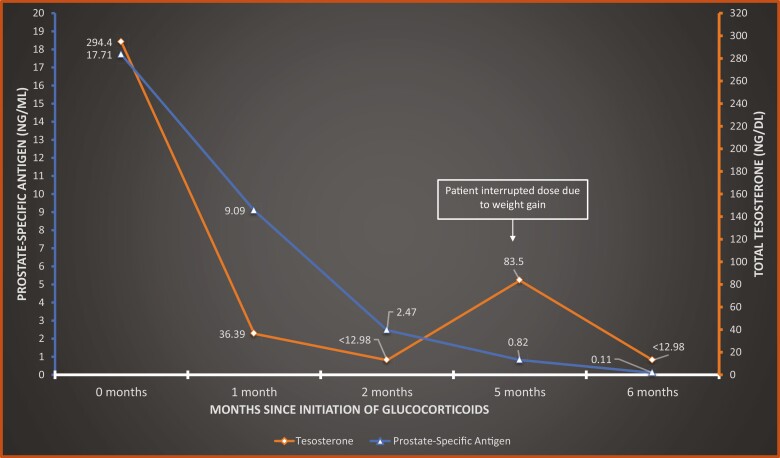
Downtrend of testosterone and prostate-specific antigen (PSA) levels following initiation of glucocorticoid therapy in patient 1 with simple virilizing congenital adrenal hyperplasia.

We present a 73-year-old man with prostate adenocarcinoma (pT2CN0M0; Gleason score 4 + 5 = 9) who underwent radical prostatectomy on initial diagnosis. He was later treated with ADT following local recurrence of the tumor with involvement of pelvic lymph nodes. Despite treatment with leuprolide, a gonadotropin-releasing hormone (GnRH) analog, he continued to have an elevated testosterone level of 294.4 ng/dL and an elevated prostate-specific antigen (PSA) level of 17.7 ng/mL. He was also noted to have incidental adrenal hyperplasia on a computed tomography (CT) imaging performed for prostate cancer staging (Fig. 1). He was referred to the Endocrinology Clinic at Orlando VA Medical Center for evaluation of persistent elevation of testosterone despite treatment with leuprolide. Given concerns about overproduction of adrenal androgens, the levels of 17-hydroxyprogesterone and dehydroepiandrosterone sulfate (DHEA-S) were measured and noted to be elevated at 10917 ng/dL (reference range, 28-250 ng/dL) and 378 mcg/dL (reference range, 5-253 mcg/dL), respectively. Consequently, an ACTH stimulation test showed significant elevation in 17-hydroxyprogesterone at baseline (19 910 ng/dL) with increase to greater than 20 000 ng/dL at 30 and 60 minutes (Table 1). The cortisol level, however, remained unchanged at 5 mcg/dL at 30 and 60 minutes, consistent with clinically occult adrenal insufficiency. Genetic testing showed biallelic mutations of the *CYP21A2* gene. The first mutation was located in intron 2 (c.293-13A/C > G), usually associated with simple virilizing or salt-wasting phenotypes of classic CAH. The second mutation was detected in the I172N sequence (c.518T > A), commonly associated with the simple virilizing phenotype. The patient had no salt-wasting features. He reported a history of infertility but no premature puberty or short stature. Based on the clinical and biochemical findings as well as genotype-phenotype association, he was diagnosed with simple virilizing CAH. The patient was treated with dexamethasone (1 mg daily) and had marked decrease in adrenal androgens, testosterone, and PSA levels (Fig. 2). He was later switched to a maintenance dose of prednisone (3 mg daily). A year after initiation of glucocorticoid therapy, he continued to have adequate control of his prostate cancer with no signs of biochemical or radiographic progression.

**Table 1.** ACTH stimulation test results in patient 1 with simple virilizing congenital adrenal hyperplasia

Test	Baseline	30 min after ACTH stimulation	60 min after ACTH stimulation	Reference range
17-hydroxypregnenolone	1101	1701	1998	< 700 ng/dL
DHEA	449	169	1384	147-1760 mcg/dL
Progesterone	8.0	22.3	17.7	< 0.4 ng/mL
17-hydroxyprogesterone	19 910	> 20 000	> 20 000	28-250 ng/dL
Androstenedione	1659	1631	1782	23-125 ng/dL
Deoxycorticosterone	< 16	< 16	< 16	< 15 ng/dL
11-deoxycortisol	62	53	54	< 110 ng/dL
Testosterone	284	281	302	190-928 ng/dL
Cortisol	5.2	4.8	5.3	2.5-22.0 ug/dL

Abbreviations: ACTH, adrenocorticotropin; DHEA, dehydroepiandrosterone.

### Patient 2

**Figure 3.**
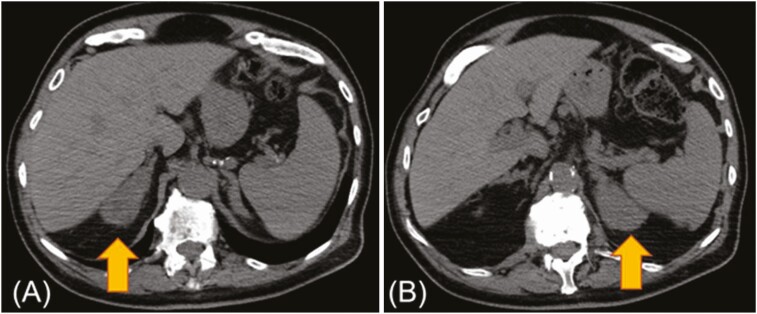
Computed tomography identified enlarged (A) right adrenal gland and (B) left adrenal gland on the axial images (arrows) in patient 2 with nonclassic congenital adrenal hyperplasia.

The second patient is an 82-year-old man with prostate adenocarcinoma (T1cNxMx, Gleason score 4 + 3 = 7) diagnosed 5 years before presentation. He initially pursued active surveillance. On surveillance, his PSA reached 14.2 ng/mL with a testosterone level of 239 ng/dL. He was treated with stereotactic body radiation therapy to the prostate in combination with leuprolide as ADT as definitive therapy in the absence of radiographic evidence of metastatic disease. However, his testosterone level remained inappropriately elevated at 87 ng/dL despite treatment with leuprolide (goal testosterone < 5 mg/dL). Consequently, the androgen receptor inhibitor bicalutamide (50 mg daily) was added to his treatment with ongoing ADT. He received 7 months of treatment with leuprolide and 4 months of combined androgen signaling inhibition with leuprolide and bicalutamide. His PSA nadir was 0.29 ng/mL, but his testosterone level remained relatively unchanged at 88.4 ng/dL. After completion of treatment with combined androgen signaling inhibition, his PSA rose to 10.86 ng/dL and testosterone to 218 ng/dL. He underwent a positron emission tomography/CT scan (PET/CT), which showed bilateral posterior iliac, right sacral, thoracic, and lumbar spine metastases as well as incidental bilateral nodular adrenal enlargement (Fig. 3). ADT was resumed with the GnRH antagonist degarelix. However, there was no improvement in his PSA (11.8 ng/mL), and testosterone remained well above castrate level (119 ng/dL). He was referred to the Endocrinology Clinic at UT Southwestern Medical Center for evaluation of adrenal androgen overproduction. A review of the patient’s history revealed premature puberty, short stature, and infertility. Further laboratory workup revealed elevated 17-hydroxyprogesterone at 4910 ng/dL (reference range, < 200 ng/dL) and DHEA-S at 312 mcg/dL (reference, < 16.2 mcg/dL) (Table 2). Based on the clinical, radiographic, and biochemical findings, the patient was diagnosed with NCCAH. He was started on treatment with the CYP17A1 inhibitor, abiraterone acetate (1000 mg daily) in combination with glucocorticoid replacement with prednisone (2.5 mg twice daily). His testosterone decreased to undetectable levels and his PSA declined to 0.41 ng/mL. One year later, an F18-fluciclovine PET/CT demonstrated interval resolution of previously seen fluciclovine-avid bone lesions, representing response to treatment. Unfortunately, his most recent evaluation showed signs of cancer progression with 2 new bone metastases in the right seventh and ninth ribs despite treatment with leuprolide, degarelix, abiraterone acetate, and prednisone, consistent with treatment failure.

**Table 2.** Laboratory evaluation before and after treatment of CAH in patient 2 with nonclassic congenital adrenal hyperplasia

Test	Pre treatment	Post treatment	Reference range
17-hydroxyprogesterone	4900		< 200 ng/dL
Androstenedione	317		40-180 ng/dL
ACTH	39 pg/mL		2.2 and 13.3 pmol/L
Cortisol	5.6		2.5-22.0 µg/dL
FSH	< 1.0		2-7 mIU/mL
LH	< 1.0		1.24-7.8 IU/L
DHEA-sulfate	312		< 16.2 mcg/dL
Aldosterone	4.0		2-9 ng/dL
Renin	3.5		2.5-45.1 pg/mL
Testosterone	117.6	< 5.0	Goal < 5.0 ng/dL
PSA	12.9	0.41	Goal < 5.0 ng/mL

Abbreviations: ACTH, adrenocorticotropin; CAH, congenital adrenal hyperplasia; DHEA, dehydroepiandrosterone; FSH, follicle-stimulating hormone; LH, luteinizing hormone; PSA, prostate-specific antigen.

## Discussion

Our 2 patients were diagnosed with CAH at an advanced age as a result of inadequate response to ADT for prostate cancer. Based on their clinical history and biochemical findings, these patients were diagnosed with simple virilizing and NCCAH, respectively.

Clinical manifestations of CAH range from mild to severe, depending on the degree of 21-hydroxylase deficiency. Males with the classic simple virilizing form typically present with early virilization (pubic hair, growth spurt, adult body odor) at age 2 to 4 years. Although CAH is one of the most common inborn endocrine disorders, the diagnosis can be missed or delayed because of subtle clinical presentation, lack of clinical suspicion, and/or awareness of the diagnosis.

Several previous observations demonstrated that diagnosis of CAH is established in fewer males compared to females, with even more pronounced discrepancy in simple virilizing patients [10-13]. In a retrospective study of 484 patients with classic forms of CAH, males were diagnosed significantly later than females with both forms (salt wasting: 26 vs 13 days [median], *P* < .001; simple virilizing: 5.0 vs 2.8 years, *P* = .03) [11]. Estimated 2 to 2.5 salt-wasting and up to 5 simple virilizing patients remain undiagnosed out of 40 expected CAH patients per year in the countries investigated in the study [11]. Clinical detection and treatment of CAH in our 2 patients were insufficient because of absent newborn screening at the time of birth of both patients and lack of clinical suspicion in the setting-provided manifestations. Newborn screening for CAH as well as greater awareness of the medical community should improve the efficacy of CAH detection and management.

Genetic testing for patient 1 revealed heterozygous, missense mutations of I172N and intron 2G (I2G) parts of the *CYP21A2* gene. According to a study of 1507 families with CAH, the frequency of I172N and I2G mutations are 8.2% and 22.9%, respectively [14]. The biallelic *I2G/I172N* mutation genotype is most prevalent in patients of European ethnicity (30/50 cases) and is predominantly associated with the simple virilizing phenotype (36/50 cases). Salt-wasting (13/50 cases) and nonclassic types (1/50 cases) were less common. These mutations result in reduced 21-hydroxylase enzyme activity to about 2% [14]. Based on the genotype-phenotype correlation, patient 1 likely had classic virilizing phenotype. He did not come to medical attention until later in his life and even then biochemical workup, rather than clinical history and clinical manifestations, prompted further workup and diagnosis. Although delineating between whether this patient has classic virilizing vs nonclassic phenotypes is inconsequential in this case, genetic testing does enrich our collective understanding of CAH. The utility of genetic testing is more paramount in younger patients for purposes of genetic counseling, fertility considerations, and for establishing diagnosis in equivocal cases [15, 16].

Nonclassic or late-onset 21-hydroxylase deficiency may present as early pubarche in school-age children, hirsutism and menstrual irregularity in young women, or there may be no symptoms. Accordingly, patient 2 with NCCAH reported early puberty and short stature. His baseline 17-hydroxyprogesterone level was moderately elevated. The diagnosis of NCCAH was made based on biochemical and clinical findings. The patient received prostate cancer treatment with combined androgen suppression with GnRH-targeted therapy plus the CYP17A1 inhibitor abiraterone in combination with prednisone. A similar case of persistent testosterone elevation despite ADT and also surgical castration was previously reported in the literature. That patient was ultimately diagnosed with NCCAH and successfully treated with hydrocortisone and prednisolone, resulting in the target castration serum testosterone level [17]. It is worth noting that our patients were born before newborn screening for CAH was introduced. Nowadays, most classic CAH cases are detected shortly after birth owing to newborn screening measures.

ADT with GnRH agonists or antagonists, or surgical castration, is recommended by the National Comprehensive Cancer Network and American Society of Clinical Oncology for treating patients with advanced prostate cancer [18]. Testosterone and PSA should both be monitored to assess for the effectiveness of ADT to suppress androgen production and cancer growth, respectively. Inadequate testosterone suppression by ADT impairs anticancer efficacy and warrants further evaluation. Several causes of persistent testosterone elevation have been described and should be considered prior to changing the treatment course. When testosterone remains elevated above castrate level (> 50 ng/dL), the first step is to repeat the test to account for possible laboratory error. Although chemiluminescent immunoassay is accurate at high levels of testosterone, it is less reliable at lower levels (< 50 ng/dL). Therefore, liquid chromatography–tandem mass spectroscopy is preferred in patients on ADT [19]. It is also recommended that the same laboratory and assay be used for testosterone monitoring to improve consistency and comparability of results [20]. The timing of the laboratory test is also important. A surge in testosterone is expected following the first injection of GnRH agonist due to temporary stimulation of the GnRH receptor. Subsequently, downregulation of testosterone production by the testicles occurs and the levels decline. Some patients may experience similar testosterone surges with readministration of GnRH agonists even after multiple injections (acute-on-chronic effect). Testosterone levels may also trend up at any point during treatment, a phenomenon known as “testosterone escape” or “breakthrough response” [18]. Therefore, it is important to take into consideration anticipated surges when interpreting the testosterone levels to avoid unwarranted changes in treatment regimen [18].

Another potential pitfall is incorrect preparation and administration of the medication, which can impair the efficacy of the medication. This can be addressed by switching the injection site and reviewing the injection procedure with the nursing staff [20]. Biodegradable lactic acid polymer microcapsules present in leuprolide injections can induce granulomatous skin reactions at the drug injection site. This has been proposed as another possible cause of hormonal escape [21]. Unfortunately, there is no treatment or preventive measure for injection-site reactions and alternative agents should be considered [22].

An uncommon case of gonadotropin-producing pituitary adenoma has been described as a cause of sustained testosterone production despite therapy with a GnRH agonist [23]. Insufficient response to GnRH agonists and higher prostate cancer mortality have also been correlated with obesity, but the mechanism is not clear [18, 24]. Molecular mechanisms involving the expression, splicing, and posttranslational modifications of the androgen receptor have been associated with resistance to ADT [25].

It is established that medical or surgical castration does not completely eliminate androgen levels and that intratumoral and adrenal androgens remain detectable [25]. While testosterone is the main circulating androgen, adrenal androgens like androstenedione and DHEA-S are also important contributors to androgen homeostasis [18]. Adrenal androgen levels are reduced by only 60% during ADT [25]. Moreover, virilizing adrenal syndromes can amplify the effects of adrenal androgens even further. Therefore, it is important to evaluate for undiagnosed adrenal etiologies of hyperandrogenemia. Based on our literature review, 2 additional cases of patients with prostate cancer with inadequate testosterone suppression despite ADT were attributed to underlying CAH [17, 26].

In patients with CAH, glucocorticoid therapy reinstates the negative feedback mechanism in the hypothalamic-pituitary-adrenal axis. The decrease in ACTH level leads to rapid decline in androstenedione and testosterone production [17]. The decrease in testosterone levels was accompanied by remarkable improvement in PSA levels in both cases presented here, though glucocorticoid therapy was used in combination with abiraterone in the treatment of the second patient. Abiraterone, a CYP17A1 inhibitor, can be used to inhibit adrenal androgen production in castration-resistant prostate cancer. By blocking CYP17A1 enzyme, abiraterone also inhibits cortisol production and can lead to mineralocorticoid excess. Therefore, abiraterone is used in conjunction with a physiologic dose of glucocorticoids to replace cortisol deficiency and prevent further escalation of ACTH stimulation and mineralocorticoid toxicity [17, 27, 28].

In conclusion, it is important to monitor serum testosterone levels in patients receiving ADT for prostate cancer and to evaluate for virilizing disorders such as milder forms of CAH in patients who show inadequate decline in androgen levels despite ADT.

## Data Availability

Data sharing is not applicable to this article as no datasets were generated or analyzed during the current study.

## Abbreviations

ACTH: adrenocorticotropic hormone
ADT: androgen deprivation therapy
CAH: congenital adrenal hyperplasia
CT: computed tomography
DHEA-S: dehydroepiandrosterone sulfate
FSH: follicle-stimulating hormone
GnRH: gonadotropin-releasing hormone
I2G: intron 2G
NCCAH: nonclassic congenital adrenal hyperplasia
LH: luteinizing hormone
LHRH: luteinizing hormone-releasing hormone
PET/CT: positron emission tomography/computed tomography
PSA: prostate-specific antigen

## Additional Information

***Disclosure Summary:*** O.H. reports research collaboration with Mayo Clinic and advisory board participation with Corcept Therapeutics and Pfizer outside the submitted work. The remaining authors have nothing to disclose.
